# Fungicide Resistance in Powdery Mildew Fungi

**DOI:** 10.3390/microorganisms8091431

**Published:** 2020-09-17

**Authors:** Alejandra Vielba-Fernández, Álvaro Polonio, Laura Ruiz-Jiménez, Antonio de Vicente, Alejandro Pérez-García, Dolores Fernández-Ortuño

**Affiliations:** 1 Departamento de Microbiología, Facultad de Ciencias, Universidad de Málaga, 29071 Málaga, Spain; avielba@uma.es (A.V.-F.); polonio@uma.es (Á.P.); laura110493@uma.es (L.R.-J.); adevicente@uma.es (A.d.V.); aperez@uma.es (A.P.-G.); 2 Instituto de Hortofruticultura Subtropical y Mediterranea “La Mayora”, Departamento de Microbiología, Campus de Teatinos, Universidad de Málaga—Consejo Superior de Investigaciones Científicas (IHSM-UMA-CSIC), 29071 Málaga, Spain

**Keywords:** cross-resistance, disease control, fitness cost, fungicide resistance, molecular mechanisms of fungicide resistance, powdery mildews, resistance development, resistance management

## Abstract

Powdery mildew fungi (Erysiphales) are among the most common and important plant fungal pathogens. These fungi are obligate biotrophic parasites that attack nearly 10,000 species of angiosperms, including major crops, such as cereals and grapes. Although cultural and biological practices may reduce the risk of infection by powdery mildew, they do not provide sufficient protection. Therefore, in practice, chemical control, including the use of fungicides from multiple chemical groups, is the most effective tool for managing powdery mildew. Unfortunately, the risk of resistance development is high because typical spray programs include multiple applications per season. In addition, some of the most economically destructive species of powdery mildew fungi are considered to be high-risk pathogens and are able to develop resistance to several chemical classes within a few years. This situation has decreased the efficacy of the major fungicide classes, such as sterol demethylation inhibitors, quinone outside inhibitors and succinate dehydrogenase inhibitors, that are employed against powdery mildews. In this review, we present cases of reduction in sensitivity, development of resistance and failure of control by fungicides that have been or are being used to manage powdery mildew. In addition, the molecular mechanisms underlying resistance to fungicides are also outlined. Finally, a number of recommendations are provided to decrease the probability of resistance development when fungicides are employed.

## 1. Introduction

**Figure 1:**
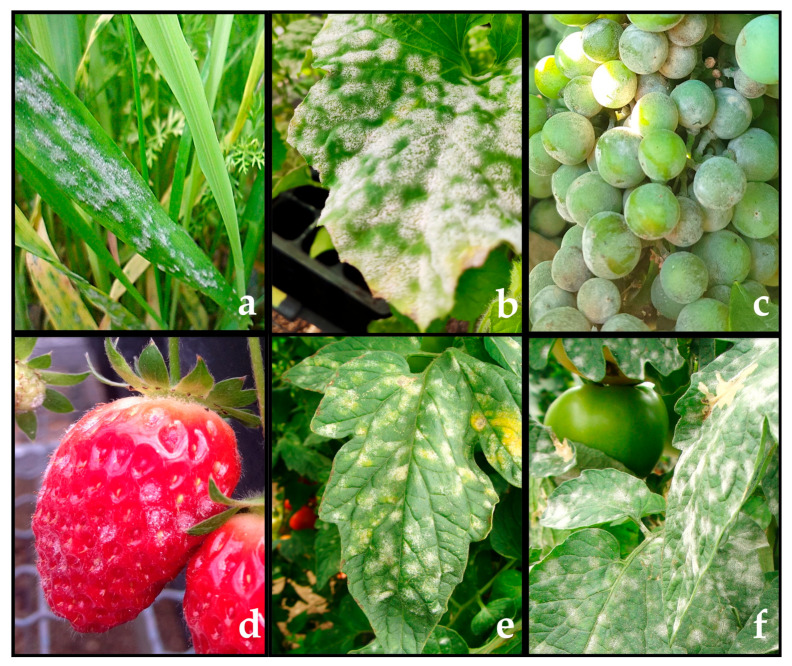
Typical powdery mildew symptoms observed on leaves and fruits of several crops. (**a**) wheat powdery mildew, caused by *Blumeria graminis* f. sp. *tritici*; (**b**) cucurbit powdery mildew, caused by *Podosphaera xanthii*; (**c**) grape powdery mildew, caused by *Erysiphe necator*; (**d**) strawberry powdery mildew, caused by *Podosphaera aphanis*; (**e**,**f**) tomato powdery mildew, caused by *Leveillula taurica* and *Oidium neolycopersici,* respectively. Picture “a” was taken from Mourad Louadfel (Homemade, Bugwood.org), pictures “b and d” were provided by the authors of this review, picture “c” was kindly provided by Beatriz López Manzanares (Instituto de Ciencias de la Vid y del Vino, Spain), picture “e” and “f” were kindly obtained from Pedro Vega (Syngenta, Spain).

Powdery mildew fungi are plant-pathogenic ascomycetes. These species comprise the Erysiphaceae family, the only family within the order Erysiphales. These fungi comprise 900 species and over 80 genera [1]. The fungi are responsible for powdery mildew diseases, which are probably the most frequent and easily recognizable plant diseases. Powdery mildew fungi cause diseases in a broad range of angiosperm hosts, including both dicot and monocot plants. Major crops, such as cereals, grapes, many fruits, vegetables and ornamental plants, are among their host plants (Figure 1). The easily recognized symptoms of powdery mildew include the presence of powdery white spots on both leaf surfaces, petioles, stems, flowers and even fruits [2]. Powdery mildews are obligate biotrophic parasites; therefore, these fungi do not directly cause plant cell death, as they need living cells to obtain nutrients and complete their life cycle [3].

**Figure 2:**
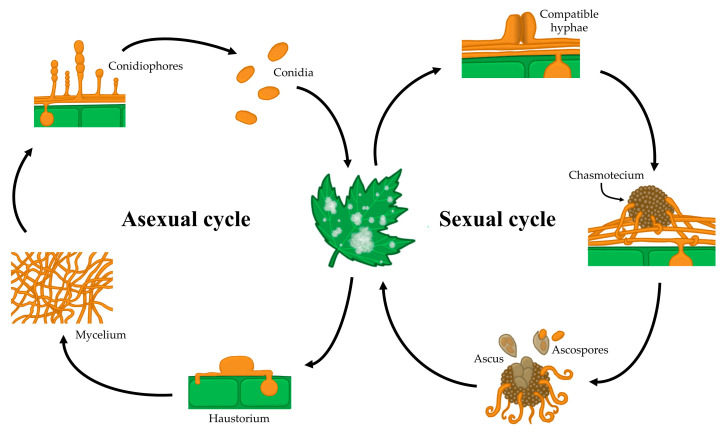
Typical life cycle of powdery mildew fungi.

In general, powdery mildew fungi show both asexual and sexual life cycles (Figure 2). The asexual life cycle begins after a conidium lands on a susceptible host plant. Then, the conidium germinates and produces a short primary germ tube that elongates to produce a specialized structure, the appressorium. This appressorium is responsible for penetrating the cuticle and the cell wall of the plant epidermal cell while remaining intact in the host plasma membrane [4]. The high turgor pressure generated in the appressorium along with the activity of a combination of lytic enzymes enables a hyphal peg to penetrate into the host epidermal cell and form a primary haustorium, which begins to take nutrients from the host [5]. If the infection is successful, the primary hyphae branch and form secondary hyphae, from which conidiophores emerge vertically, producing a variable number of conidia (one or more) depending on the genus [6,7,8,9,10]. Some days after infection, this epiphytic fungal growth results in typical disease symptoms that are visible to the naked eye as white colonies on plant surfaces.

In the case of sexual reproduction, two compatible mating type hyphae merge and form a fruiting body called a chasmothecium, which contains, according to the genus, one or more ascus bearing the sexual spores or ascospores [6]. Chasmothecia are considered to be overwintering and oversummering sources of inoculum [7,9,10]. Although the outbreak of the disease caused by ascospores has not been fully elucidated, is thought to resemble that of conidia [11]. For some species, such as the grape powdery mildew *Erysiphe necator*, chasmothecia have high epidemiological importance [12]. For other species, such as the cucurbit powdery mildew *Podosphaera xanthii*, chasmothecia are rarely observed or have never been observed in the field, and therefore, the epidemiological relevance of the sexual life cycle of this pathogen has not been determined [8,13].

At present, the management of powdery mildew diseases is based on two primary control practices: the use of resistant varieties and the application of fungicides. For some crops, commercial varieties and breeding lines with resistance to powdery mildew are available [14,15]; however, the rapid development of new races of pathogens hinders the management of this disease by resistance breeding [8]. Safer alternatives to chemicals, including inorganic, organic and biological control products, are also available [16]; however, in practice, the application of fungicides continues to be the principal tool for the management of powdery mildews in many crops, which often leads to the development of resistance to the most frequently employed mildewcides [17]. The purpose of this review is to outline the current body of knowledge on fungicide resistance in powdery mildew fungi. Cases of resistance to the chemical classes of fungicides that are used for the control of powdery mildew fungi in different crops and countries are described. In addition, the findings concerning the cross-resistance problems, the fitness costs and the molecular mechanisms associated with resistance to fungicides are also presented. Finally, some recommendations concerning resistance management are also presented.

## 2. Fungicides, the Main Anti-Powdery Mildew Tools

Although more suitable and environmentally friendly alternatives have been explored, in practice, the application of fungicides remains the primary strategy for the control of powdery mildew diseases, with large investments being made every year in these chemical products [18]. Several fungicides from different chemical classes are currently available for the management of powdery mildew (Table 1), including 10 multisite fungicides belonging to the groups chloronitriles, dithiocarbamates, phthalimides and quinoxalines (Fungicide Resistance Action Committee (FRAC) codes M05, M03, M04 and M10, respectively); 75 site-specific fungicides, including the amines groups, which target sterol biosynthesis (also known as morpholines; four active ingredients (a.i.); FRAC code 5), the anilino pyrimidines, which inhibit amino acid and protein synthesis (three a.i.; FRAC code 9), the aryl-phenyl-ketones, which affect cytoskeleton and motor protein function (two a.i.; FRAC code 50), the aza-naphthalenes, which block signal transduction (two a.i.; FRAC code 13), the demethylation inhibitors, which also inhibit sterol biosynthesis (DMI fungicides; 29 a.i.; FRAC code 3), the dithiolanes, which affect membrane integrity or function (one a.i.; FRAC code 6), the hydroxy-(2-amino) pyrimidines, which target nucleic acid metabolism (one a.i.; FRAC code 8), the ketoreductase inhibitors, which also inhibit sterol biosynthesis (one a.i.; FRAC code 17), the methyl-benzimidazole carbamates, which block mitosis (MBC fungicides; five a.i.; FRAC code 1), the phenylpyrroles, which also affect signal transduction (one a.i.; FRAC code 12), the polyoxins, which inhibit chitin biosynthesis (one a.i.; FRAC code 19), the quinone outside inhibitors (QoI fungicides; 10 a.i.; FRAC code 11), the succinate dehydrogenase inhibitors (SDHI fungicides; 11 a.i.; FRAC code 7) and the uncouplers of oxidative phosphorylation (three a.i.; FRAC code 29) all three of which affect cellular respiration; finally, three fungicides with unknown modes of action, which belong to the cyanoacetamide-oxime, phenyl-acetamide and thiazolidine groups (FRAC codes 27, U6, and U13, respectively).

**Figure 3:**
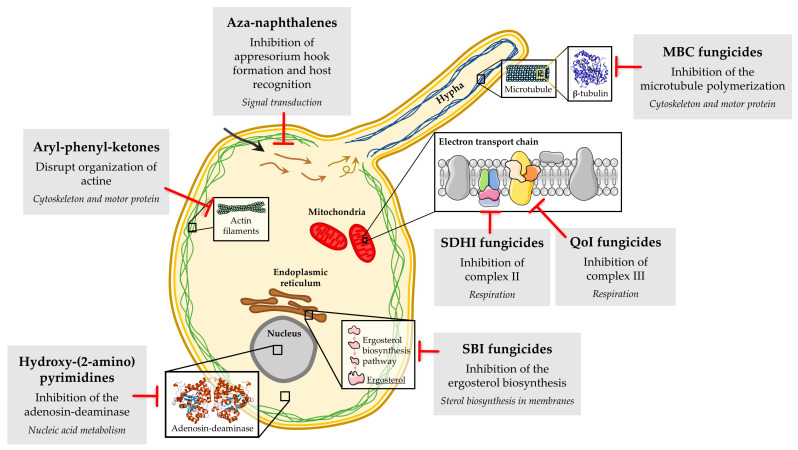
Biochemical mode of action of the main classes of fungicides with resistance described in powdery mildew fungi.

Unfortunately, chemical control of powdery mildew fungi has been hindered by the emergence of fungicide-resistant isolates. Fungicide resistance in powdery mildew is an important problem that causes economically important losses to growers around the world every year, especially when site-specific fungicides are used. Two types of fungicide resistance have been described. The so-called “qualitative” resistance shows an abrupt loss of effectiveness, with the isolates of fungal population being divided into two separate groups, the sensitive and the resistant isolates. The so-called “quantitative” resistance manifests a gradual decline in disease control, with variable degrees of fungicide resistance being observed in the isolates of the fungal population [17,19]. Several molecular mechanisms have been associated with resistance problems to site-specific fungicides in powdery mildew fungi (Figure 3), with the primary one being target site-based, involving mutations in genes encoding target proteins, which result in peptide sequence changes that prevent fungicide binding. Examples include resistance to DMI, MBC, QoI and SDHI fungicides. In addition, the overexpression of the target gene due to the occurrence of mutations in promoter sequences and the participation of genes encoding drug efflux transporters (e.g., ATP-binding cassette) have also been described in DMI resistance [20,21,22]. For other fungicides, such as aryl-phenyl-ketones, aza-naphthalenes, and hydroxy-(2-amino) pyrimidines, although the primary mode of action is known, the mechanisms involved in resistance have not been fully elucidated to date [23,24,25].

The FRAC and the European and Mediterranean Plant Protection Organization (EPPO) have classified powdery mildew species depending on the risk of the pathogen developing resistance to fungicides under specific agronomic conditions [26]. In this regard, *Blumeria graminis* (wheat and barley powdery mildew)*, E. necator* (powdery mildew of grape) and *P. xanthii* (cucurbit powdery mildew) are considered to be pathogens with high risk of resistance development because they show short disease cycles per season, their dispersal through conidia over time and space is high, and they have evolved resistance to several classes of fungicides after a few years of product use. These characteristics make these pathogens serious threats to the commercial success of site-specific fungicides. Other species, such as *Leveillula taurica* and *Oidium neolycopersici* (tomato powdery mildews)*, Sphaerotheca macularis* (powdery mildew of several hosts) and *Sphaerotheca mors-uvae* (gooseberry powdery mildew)*,* possess medium risk, meaning that resistance is not a major problem or has been slow to develop, and for this reason, in commercial practice, fungicide resistance has not created major disease control problems. For other powdery mildews, such as *Podosphaera leucotricha* (powdery mildew of apple), resistance against only a small number of chemical classes has been observed; therefore, this species is considered to be a low-risk pathogen with low importance in commercial market terms [26].

## 3. Current Fungicide Resistance Status in Powdery Mildew Fungi

### 3.1. Resistance to Aryl-Phenyl-Ketones in B. graminis f. sp. hordei, B. graminis f. sp. tritici, E. necator and P. xanthii

The aryl-phenyl ketones are classified in FRAC code 50 (and reclassified from U8 in 2018). Two chemical groups form this chemical class: the most widely used benzophenone, metrafenone, and the benzoylpyridine pyriofenone (Table 1). Metrafenone and pyriofenone were introduced to control the powdery mildew diseases of wheat, barley, grapes and vegetables in Europe and Japan in 2006 and 2014, respectively. No reports of control failure were documented until 2009 and 2017 (Table 2).

A limited number of studies have investigated metrafenone sensitivity in powdery mildew fungi. The first study that reported low levels of metrafenone resistance was conducted in *B. graminis* f. sp. *tritici* in 2009 [27]. In that study, 2509 airborne isolates collected from different regions of Europe were analysed. Only 84 isolates exhibited a metrafenone sensitivity reduction in laboratory studies without compromising control failures in the field; however, eight isolates were uncontrolled at the recommended field dose. In another study, a low proportion of metrafenone-resistant isolates were described, and only 1% of the isolates that were analysed presented values of the effective concentration of fungicide inhibiting pathogen development at 50% (EC_50_) above 10 mg/L of the formulated product [28]. In addition, these authors observed that, in competitive assays, isolates with the sensitive phenotype had a higher fitness than the resistant ones, understanding fitness as the efficiency of important physiological and biochemical processes [28,29,30]. A few years later, a new monitoring study conducted in Europe showed significant levels of resistance to metrafenone in *B. graminis* f. sp. *tritici*, with almost one-third of the isolates showing moderate (27%) or high (1%) resistance [31].

Regarding the grapevine powdery mildew *E. necator*, the first incidence of metrafenone resistance was reported in 2013 after a study conducted in northern Italy [32]. In that study, only 2 out of 13 isolates were sensitive to metrafenone, with the rest of the isolates exhibiting a variable range of sensitivity (125–1250 mg/L of the formulated product) to this fungicide. In the same study, cross-resistance problems were also documented, with metrafenone-resistant isolates exhibiting cross-resistance to pyriofenone [32]. Later, a survey conducted in Europe in 2016 reported that 17.5% of the *E. necator* population was resistant to metrafenone [31].

Only recently has a study investigated the sensitivity of the cucurbit powdery mildew pathogen to the benzoylpyridine pyriofenone. In that study, which was conducted in Japan, 89 out of 122 *P. xanthii* isolates were highly resistant to this fungicide, having EC_50_ values of >1000 mg/L of the formulated product and a resistance factor (RF, calculated by dividing the mean of EC_50_ values of the resistant population by the mean of EC_50_ values of the sensitive population) of >1840 [33].

The molecular mechanism that confers resistance to aryl-phenyl-ketones has not been fully elucidated. To date, studies performed in *B. graminis* f. sp. *tritici* and f. sp. *hordei* have revealed that metrafenone affects spore germination, appressorial formation and penetration, surface hyphal morphology and sporogenesis, at least in these powdery mildew fungi. This active ingredient likely affects a pathway regulating the organization of the actin cytoskeleton, causing the loss of cell polarity (Figure 2) [24,34].

### 3.2. Resistance to Aza-Naphthalenes in B. graminis f. sp. hordei, B. graminis f. sp. tritici, E. necator and P. xanthii

The aza-naphthalenes fungicides (FRAC code 13) were specifically designed to control powdery mildew diseases [35,36]. At present, two active ingredients are registered, quinoxyfen and proquinazid (Table 1).

Quinoxyfen was registered in Europe and in the US in 1996 and in 2007, respectively. From 1995 to 2000, this active ingredient was effective [37]; however, quinoxyfen resistance issues were reported in *B. graminis* f. sp. *tritici* in northern Germany in 2003 and in France between 2003 and 2005 [38] (Table 2). Despite these reports, almost twenty years after the registration of quinoxyfen, there are no serious problems in the control of this powdery mildew species with this active ingredient [39]. High levels of sensitivity to the other aza-naphthalene were found in wheat powdery mildew, with proquinazid equally controlling *B. graminis* f. sp. *tritici* isolates that were sensitive and resistant or less sensitive to other chemical groups [40]. On the other hand, a study developed in *B. graminis* f. sp. *hordei* showed that, in laboratory studies, the quinoxyfen-resistant isolates exhibited a fitness penalty, and they only survived when this fungicide was applied [41]. Perhaps the good performance of quinoxyfen against cereal powdery mildews is due to the strong fitness cost exhibited by quinoxyfen-resistant mutants in these powdery mildew species.

The aza-naphthalene resistance situation was not the same for the grapevine powdery mildew pathogen *E. necator* (Table 2). In a quinoxyfen resistance monitoring study conducted in Europe and South Africa in 2002, 32 of the 50 isolates analysed showed a decrease in sensitivity; however, some discrepancies regarding the EC_50_ values were obtained for the resistant isolates regarding spore germination and leaf disk assays [42]. Quinoxyfen resistance problems were also described in the US, specifically in studies conducted in vineyards in western New York (from 2010 to 2013) and Virginia (during 2013) [43,44,45,46]. By contrast, a few years later, another study investigated the evolution of resistance in the same Virginia field to different chemical classes, including quinoxyfen, determining that this aza-naphthalene provided better control did the other fungicides [47]. In any case, cross-resistance problems in this chemical class have been reported in *E. necator* [40]. In that study, the authors concluded that the risk of the development of resistance to proquinazid might be influenced by the use of quinoxyfen and recommended that proquinazid and quinoxyfen be managed together for optimal resistance management.

Regarding the cucurbit powdery mildew *P. xanthii*, a sharp shift to resistance was observed in the American cucurbit fields over a period of years. Quinoxyfen provided good control against cucurbit powdery mildew during the period 1996–2000; however, the sensitive values changed over time, and some isolates collected from New York were able to grow at 10 mg/L of the formulated product in 2004 [48,49] (Table 2). In 2007, 62% of the isolates analysed from Georgia and New York were able to grow at 10–15 mg/L of commercial quinoxyfen [50]. This trend was extended, and a few years later, 24% and 4% of *P. xanthii*-resistant isolates collected from New York were able to grow at 40 mg/L and 80 mg/L of the formulated product, respectively [51]. Finally, in 2015, 21 out of 57 *P. xanthii* isolates collected from the same state were able to tolerate 200 mg/L of the commercial fungicide quinoxyfen [52].

In relation to the molecular mechanisms that confer resistance to the aza-naphthalene class, several studies have been conducted with quinoxyfen and proquinazid in *B. graminis* f. sp. *hordei*. Regarding quinoxyfen, studies have shown that this active ingredient increases the levels of several kinases (*cpka*, *pkc1* and *pkc*-*like*) and slows their degradation, with their decay being required for appressorium hook formation [53]. In addition, quinoxyfen also interfered with the signalling events performed by the Ras G-protein [53]. A few years later, another study determined that quinoxyfen disrupted the perception/signalling events involved in host recognition and germination in the same pathogen [54]. In addition, the serine esterase activity of the extracellular matrix of the conidia also appeared to be affected, with the serine esterase CUT-1 being inhibited in the wild type and overexpressed in the resistant isolates [54]. Regarding proquinazid, signal transduction seemed to be affected but in a different manner from quinoxyfen, indicating that these two fungicides could have different modes of action [25] (Figure 2). Therefore, interference with signal transduction pathways appears to be the mode of action of aza-naphthalenes, but the precise targets of quinoxyfen and proquinazid have not been identified to date.

### 3.3. Resistance to Hydroxy-(2-amino) Pyrimidines in B. graminis f. sp. hordei, Golovinomyces cichoracearum and P. xanthii

Another class of fungicides that has a specific effect against powdery mildew fungi is the hydroxy-(2-amino) pyrimidines (FRAC code 8). Currently, bupirimate is the only active ingredient registered for powdery mildew control, but in the past, dimethirimol and ethirimol were also available (Table 1).

Dimethirimol was registered in 1968 and was widely employed by growers to control cucurbit powdery mildew in the late sixties; however, a study that reported that some *P. xanthii* isolates were able to tolerate even 100-fold higher concentrations than the sensitive isolates was published in Germany two years after the registration of dimethirimol [55] (Table 2). Furthermore, the persistence of resistance to dimethirimol was observed in *P. xanthii* 10 years after the withdrawal of this fungicide in the Netherlands [56].

Ethirimol was also marketed in 1968 for the control of barley powdery mildew. The first case of resistance was reported in the UK in 1971, with *B. graminis* f. sp. *hordei* isolates being observed to tolerate 10–100 times higher concentrations of ethirimol than the sensitive isolates [57] (Table 2). In 1979, ethirimol-resistant *B. graminis* f. sp. *hordei* isolates were still documented in the same country, and the results of this study showed that the resistance was stable, at least during one season [58]. However, a competitive study that included an ethirimol sensitivity bioassay, genotype stability assay, and in vivo assays testing the virulence and germination of the isolates in different varieties of barley with various ethirimol treatments showed that the ethirimol-resistant isolates had lower fitness in comparison to the sensitive isolates [59].

Bupirimate was the third hydroxy-(2-amino) pyrimidine introduced for the control of powdery mildew in several crops in 1975. Cases of resistance to this fungicide have only been described in cucurbit powdery mildew species. The first case was described in *P. xanthii* in isolates collected from Greece and Australia in 1986 and 1988, respectively [60,61] (Table 2). The Greek isolates were able to tolerate the dose of 125 mg/L of the commercial bupirimate [60] when two out of six Australian isolates were able to develop disease on melon plants treated at 200 mg/L of the formulated product [61]. In 1990, two studies performed in Egypt in *G. cichoracearum* reported a decreased sensitivity after multiple applications of this fungicide and cross-resistance to dimethirimol [62,63] (Table 2). Years later, a study performed in Spain reported two highly resistant *P. xanthii* isolates with a minimal inhibitory concentration (MIC) value for bupirimate of 1.000 mg/L of the formulated product, which was 2.6-fold higher than the maximum dose (375 mg/L) recommended to control the disease in the field [64].

The primary mode of action of hydroxy-(2-amino) pyrimidines seems to be interfering with nucleic acid metabolism by blocking adenosine-desaminase [23,65] (Figure 2). To date, only one study has been developed to clarify the molecular mechanism of action of hydroxy-(2-amino) pyrimidines. In this study, it was observed that ethirimol inhibited adenine incorporation into RNA during appressorium formation in the barley powdery mildew [65]. Similar to the lack of knowledge regarding the mode of action of these fungicides, the molecular mechanism of resistance to hydroxy-(2-amino) pyrimidines has not been elucidated to date.

### 3.4. Resistance to MBC Fungicides in E. necator, G. cichoracearum, P. xanthii and Sphaerotheca pannosa

MBC fungicides (FRAC code 1) were first introduced in the market in the late 1960s and 1970s for the control of many plant diseases, including powdery mildews. Currently, several active ingredients classified into two groups comprise this class of fungicides (Table 1): benzimidazoles (benomyl, carbendazim, fuberidazole and thiabendazole) and thiophanates (thiophanate and thiophanate-methyl).

Resistance to the MBC fungicide benomyl was first described in the US in 1967 in the cucurbit powdery mildew *P. xanthii* [66] (Table 2). Soon after that study was performed, the shift of MBC resistance increased rapidly up to 68–100% in this powdery mildew in the same country [67,68]. *P. xanthii* isolates that were able to grow at 50 and 250 mg/L of commercial benomyl were also detected in high frequencies in Australia [61,69]. In Europe, the situation was highly similar. In several surveys performed from 2001 to 2007 in the Czech Republic, benomyl was not effective in controlling any cucurbit powdery mildew, neither were *P. xanthii* or *G. cichoracearum*, with the frequency of resistant isolates being higher than 90% in most of the years sampled [70,71,72,73] (Table 2). A similar strong selection with very high frequencies of resistance (>90%) to thiophanate-methyl was also observed in Austria, France, Italy, Spain and the Czech Republic. Cucurbit powdery mildew isolates presenting MIC values of >1000 mg/L of the formulated product were observed very often; this concentration was considerably higher than that recommended and sprayed in cucurbit fields [64,71,73,74] (Table 2).

MBC-resistant isolates were also detected in other powdery mildew species. For example, the rose powdery mildew *S. pannosa* was also uncontrolled, despite the benomyl sprays, in Ontario (Canada) during 1974 and 1975 [75] (Table 2). When benomyl was registered in the US for the control of the grapevine powdery mildew *E. necator*, it provided good control during the period of 1973–1976; however, a few years later, loss of disease control was also described on grapes from western New York [76] (Table 2). In addition, benomyl-resistant isolates were still detected in California after two decades of continued use of this fungicide [77], indicating that resistant isolates can persist in fields for many years. With regard to thiophanate-methyl, in a study performed in the US between 2005 and 2007, sixty-one *E. necator* isolates collected from 23 locations in Virginia were determined to be resistant to this active ingredient [46] (Table 2).

Even though the mechanism of MBC resistance is well documented in several plant pathogens, very little has been reported regarding powdery mildews. The main mechanism of resistance described to this class of fungicides is due to several point mutations in the gene encoding β-tubulin, affecting the microtubule dynamic [78] (Figure 2). The most common amino acid changes described in the literature for several plant pathogens were the E198A/K/V or F200Y substitutions [79]. Only one of these amino acid changes, E198A, was reported in 11 thiophanate-methyl-resistant isolates of *P. xanthii* collected from several cucurbit growing areas in Spain from 1996 until 2008 [80] (Table 3). MBC-resistant isolates of *P. xanthii* carrying the same amino acid change in β-tubulin were still observed at high levels in Spain in 2018 [74]. These results indicate that resistance to MBC fungicides is widespread and prevalent in cucurbit fields in this country and probably in other locations where these fungicides have been used extensively.

### 3.5. Resistance to Phosphorothiolates in P. xanthii

A limited number of studies have investigated the sensitivity of powdery mildews to phosphorothiolates, a class of fungicides that are not currently available for powdery mildew control. Pyrazophos was the first active ingredient of this class registered to control cucurbit powdery mildew in 1970; however, the first control failure of pyrazophos was reported in the Netherlands a few years later [81] (Table 2). In that study, 32% and 9% of the *P. xanthii* isolates that were analysed were able to grow at 1 or 3.2 mg/L of the formulated product, respectively, 3.3- and 10.6-fold higher than the tolerance of sensitive isolates. Fortunately, the resistant isolates appeared to have fitness penalties, and after a year without using pyrazophos, sensitivity levels returned to baseline levels [81]. A study conducted in the same country in 1981 and 1983 observed that 13 out of 194 *P. xanthii* isolates were resistant to this fungicide with EC_50_ values > 60 mg/L of the pure active ingredient; however, in this case, resistance seemed to be stable over a period of years [56]. A region-wide resistance monitoring survey was conducted years later in Australia, reporting that 21% of the *P. xanthii* isolates analysed were resistant to pyrazophos [69].

To the best of our knowledge, no prior studies have investigated resistance to other phosphorothiolates or the molecular mechanisms of resistance to pyrazophos in powdery mildew fungi.

### 3.6. Resistance to QoIs in B. graminis f. sp. hordei, B. graminis f. sp. tritici, Erysiphe betae, E. necator, Leveillula taurica, Podosphaera leucotricha and P. xanthii

Quinone outside inhibitors (FRAC code 11) are an important class of fungicides that are widely used in agriculture to control powdery mildews in economically important crops, such as cereals, grapes or cucurbits. The registration and commercialization of the first QoIs were undertaken in 1992; however, resistance to the fungicides azoxystrobin, famoxadone, fenamidone and kresoxim-methyl was documented in the cucurbit powdery mildew in different parts of the world a few years later [82,83] (Table 2). More reports on QoI resistance in *P. xanthii* were also made in Spain and the US [84,85]. Thirty-two percent of the *P. xanthii* isolates collected from several locations in Spain had MIC values >500 mg/L for commercial azoxystrobin, trifloxystrobin and kresoxim-methyl [84] (Table 2), whereas 80% of the American isolates were able to grow at 100 mg/L commercial trifloxystrobin [85]. In both cases, cross-resistance to all the QoIs tested was observed [84,85]. Similar results were described in the Czech Republic, with isolates of *P. xanthii* and *G. cichoracearum* developing cucurbit powdery mildew disease at commercial azoxystrobin doses of above 500 mg/L [72,73] (Table 2). In regard to the stability of QoI resistance in the cucurbit powdery mildew, a Japanese study revealed that under greenhouse conditions, the resistance to azoxystrobin seems to be unstable, *P. xanthii* isolates resistant to this fungicide two-and-a-half years after not being applied in the field [86]; however, opposite results were found under laboratory conditions, with *P. xanthii* isolates maintaining high levels of azoxystrobin resistance after three years of subculturing without this fungicide [86].

Cases of resistance to QoIs were also documented in wheat and barley powdery mildews. Resistance frequencies as high as 70–90% to trifloxystrobin were reported in Germany at the end of the 1990s in *B. graminis* f. sp. *tritici* [87] (Table 2) but also in France (14%), Belgium (30%), Denmark (44%) and the UK (5%) [87]. In addition, it was also reported that resistant isolates showed greater fitness than sensitive isolates, with stable levels of resistance being observed over 3 years [87]. Cross-resistance to trifloxystrobin and kresoxim-methyl was also observed [87]. Resistance to the QoI fungicides famoxadone and fenamidone has also been described in this species [83] (Table 2). Regarding *B. graminis* f. sp. *hordei*, resistance to kresoxim-methyl and trifloxystrobin was documented in Europe in 2003 [88] (Table 2).

In relation to the grape powdery mildew *E. necator*, the first cases of QoI resistance were described in the US. The first study observed that 2% of the 256 isolates collected from New York in 1999 were able to tolerate 2 mg/L of technical grade azoxystrobin [89] (Table 2). A second study tested the sensitivity of 35 isolates to trifloxystrobin. These isolates, which were collected from California between 1999 and early 2000, presented EC_50_ values of 12.8 mg/L of the pure active ingredient [90] (Table 2). A few years later, highly trifloxystrobin-resistant isolates (EC_50_ > 100 mg/L of the formulated product) were also found in high frequencies in Michigan (62%), Virginia (90%) and North Carolina (82%) [46,91,92]. Azoxystrobin- and trifloxystrobin-resistant isolates have been described in India and New Zealand, respectively [93,94]. Unfortunately, another study reported that the stability of QoI resistance in *E. necator* seemed to persist even two years after the absence of exposure to QoI fungicides [95].

A similar strong selection for resistance to QoIs has been observed in other powdery mildew species. Pyraclostrobin and trifloxystrobin-resistant isolates of the sugar beet powdery mildew *E. betae* (synonym of *E. polygoni*) were documented in the US in 2011 [96] (Table 2) and isolates resistant to pyraclostrobin were observed in Scandinavia in 2015, 2017 and 2018 [97]. Some studies conducted with the apple powdery mildew *P. leucotricha* reported a decrease in sensitivity in fields treated with trifloxystrobin in Germany, India and the UK [98] (Table 2). One of the trifloxystrobin-resistant isolates detected in this study was able to grow at a fungicide concentration 10-fold higher than the recommended field dose. In addition, after 50 generations, two isolates still showed a reduced sensitivity to QoI fungicides [98]. Finally, regarding the tomato powdery mildew *L. taurica*, 48% of the isolates analysed in the US in 2015 were resistant to azoxystrobin [99] (Table 2).

The QoI fungicides act by binding the outer quinol oxidation site (Qo site) of the cytochrome bc1 complex, interfering with the electron transport chain of the respiration pathway and leading to an energy deficiency in fungal cells by halting the production of ATP [100] (Figure 2). Resistance to QoI is qualitative and is conferred by mutations in the mitochondrial cytochrome b (*cytb*) gene. Mutations affecting sensitivity have been described in two regions corresponding to amino acid positions 120–155 and 255–280 of the encoded protein, with the amino acid change from glycine to alanine at position 143 (G143A) being the most commonly reported (Table 3). This mutation has been detected in QoI-resistant isolates of *B. graminis* f. sp. *hordei* [88], *B. graminis* f. sp. *tritici* [83,101,102,103,104,105], *E. betae* [96,97], *E. necator* [46,91,92,93,94,95], *L. taurica* [99], *P. leucotricha* [98] and *P. xanthii* [82,83,106]. This mutation does not seem to affect the activity of the enzyme, since resistant isolates can persist for several years in the absence of the application of QoI fungicides [83,87,94,98,104]. A phenomenon called heteroplasmy, which is the coexistence of sensitive (G143) and resistant (A143) cytb alleles in the same fungal cell, appears to govern QoI resistance in some powdery mildew species, such as *L. taurica* [99], *P. leucotricha* [98] and *P. xanthii* [106]. However, other authors have detected the presence of an intron of 2.438 bp directly after codon 143 in *P. leucotricha* isolates collected from Austria, Belgium, France, Germany, Italy, the Netherlands, Spain and the UK [107]. This intron hindered the development of resistance to QoI by preventing the alteration of amino acids at this position, since any alteration at this position would lead to the creation of a dysfunctional Cytb protein [107].

### 3.7. Resistance to SBI Fungicides, Amines and DMIs in B. graminis f. sp. hordei, Blumeria graminis f. sp. tritici, E. necator, G. cichoracearum, Podosphaera aphanis and P. xanthii

SBIs are one of the most important families of fungicides, with 30% of the products of the fungicide market belonging to this class [108]. Four chemical groups, namely, amines or morpholines (FRAC code 5), ketoreductase inhibitors (KRIs) (FRAC code 17), SBI class IV (FRAC code 18) and demethylation inhibitors (DMIs) (FRAC code 3), form this extensive family of fungicides with 48 active ingredients [78]. The amines, the KRIs and the DMIs are the groups currently registered for powdery mildew control (Table 1).

The first cases of amine (tridemorph, fenpropimorph and fenpropidin) and DMI (triadimenol, triadimefon, propiconazole, diclorobutrazol, nuarimol and procloraz) resistance were described in the barley powdery mildew *B. graminis* f. sp. *hordei* in various European regions at the end of the 1970s and the beginning of the 1980s [58,109,110,111,112] (Table 2). In later studies, it was found that the levels of resistance to fenpropimorph, fenpropidin and triadimefon did not compromise their field efficacy [113]. However, for triadimenol, 58.9% of the *B. graminis* f. sp. *hordei* isolates collected from barley seedlings were resistant at a field dose of 2.5 kg/seed [114]. During the 1990s, a reduced effect of amines (fenpropimorph and fenpropidin) and several DMIs (cyproconazole, epoxiconazole, fenpropimorph, flutriafol, propiconazole, tebuconazole, and triadimenol) were reported in the same pathogen in different parts of Europe, including Hungary, England and Scotland [115,116,117,118,119] (Table 2). Triadimenol resistance was also observed in 2013 in Brazilian farms, with this fungicide exhibiting only 26.1% disease control [120]. Regarding the wheat powdery mildew *B. graminis* f. sp. *tritici*, the first cases that reported a reduction in sensitivity to several DMIs were made in Germany and the Netherlands between 1981 and 1984 [112,121] (Table 2). In addition, a lower fitness of the resistant isolates was also described [112]. Over the years, several cases of reduced sensitivity to the DMIs triadimefon and triadimenol were reported in Canada, China and Europe [119,122,123,124,125] (Table 2). In Brazil, the sensitivity to triadimenol also decreased in *B. graminis* f. sp. *tritici*, with only 18.1% of the disease being controlling in wheat seeds [126].

The cucurbit powdery mildew species *P. xanthii* and *G. cichoracearum* also developed resistance to DMIs. Control failure of *P. xanthii* with imazalil and triforine was reported in the Netherlands a few years after their registration [127,128] (Table 2). Although more cases of reduced sensitivity to amine tridemorph and DMIs (fenarimol, imidazil, triadimefon and triforine) were reported during the 1980s, the control of the disease in the field appeared to be achieved [61,128,129] (Table 2). In the 1990s, triadimefon-resistant *P. xanthii* isolates able to develop disease at 200 mg/L of the formulated product in in vitro bioassays were also described in the US [130]. Another study conducted in the same country described a sharp increase in the percentage of resistant isolates from 0 to 96% in a short period of time and only after two triadimefon applications [67]. In addition, *P. xanthii* isolates able to grow at 50 mg/L of technical grade triadimefon and tolerating 5 mg/L and 20 mg/L of technical grade myclobutanil and commercial propiconazole, respectively, were also found in nontreated fields in the US [68] (Table 2). The expansion of DMI resistance has continued over the years, and *P. xanthii* and *G. cichoracearum* DMI-resistant isolates have also been reported in several parts of the world, such as Australia [61,69], Greece [60], Japan [131], South Korea [132] (Table 2), Spain [133], the Czech Republic [73] and the US [134,135]. *P. xanthii* DMI-resistant isolates did not exhibit any fitness penalty, exhibiting stable resistance in the absence of fungicide over the years [128,136]. In addition, multiple resistance to several DMIs and non-related fungicides, such as boscalid and quinoxyfen, have also been reported in the cucurbit powdery mildew in the Netherlands and US [50,128,137].

The first instance of *E. necator* resistance to DMIs, such as fenarimol, myclobutanil and tridiamefon, was reported in 1985 and confirmed in 1986 and 1990 in the US, specifically in California [138] (Table 2). During the 1990s, resistance to DMIs in *E. necator* was reported in France, Portugal and New York (US) [139,140] (Table 2). In the following years, more cases of declining sensitivity emerged in different parts of the world, such as Africa (resistance to triadimenol, penconazole and flusilazole [141] (Table 2); Australia (triadimenol and fenarimol) [142]; Austria (triadimefon, triadimenol, myclobutanil and penconazole) [143]; Canada (myclobutanil) [144]; India (myclobutanil) [145]; Iran (penconazole and hexaconazole) [146] (Table 2); New Zealand (myclobutanil and penconazole) [94]; and US (myclobutanil, spiroxamine, tebuconazole, triflumidazole, fenarimol and triadimefon) [77,91,147] (Table 2). Although some studies have reported *E. necator* isolates with non-existent or very low levels of cross-resistance to DMIs [139], other studies have shown that, in fields where triadimenol or triadimefon had been sprayed, the resistance of *E. necator* to those fungicides increased, as well as to myclobutanil and fenarimol, although at lower levels [140].

Resistance to the group of the DMI fungicides triazoles was also detected in the strawberry powdery mildew *P. aphanis* (previously known as *S. macularis*) in a study performed in France [148] (Table 2). Twenty-four isolates collected from strawberry fields that had been exposed to penconazole or myclobutanil were tested in sensitivity bioassays using leaf discs incubated with different concentrations of the fungicides. Myclobutanil-resistant isolates with EC_50_ values ranging from 1.7 to 14.67 mg/L of the formulated product and penconazole-resistant isolates with EC_50_ values of 1.9 to 4.2 mg/L of the commercial fungicide were obtained [148].

To elucidate the molecular mechanisms of DMI resistance in *B. graminis* f. sp. *hordei*, the *cyp51* gene was amplified and sequenced in several studies (Table 3). The amino acid change Y136F explained the resistance of the isolates collected from the European, Indian and Israeli barley fields treated with triadimenol [149]. The change in K147Q was also observed in *B. graminis* f. sp. *hordei* isolates with reduced sensitivity to triadimenol in the UK [150]. In Western Australia, the amino acid change S509T in combination with Y136F was highly correlated with epoxiconazole and tebuconazole resistance in the same pathogen [151,152,153]. Regarding the wheat powdery mildew *B. graminis* f. sp. *tritici*, the amino acid changes Y136F and K147Q were also correlated with high levels of resistance to the group of the DMI fungicides triazoles in the UK [150] (Table 3). The grape powdery mildew *E. necator* was not an exception, and the amino acid change Y136F was also observed in isolates exhibiting high levels of resistance to triadimenol [154]. The same amino acid change was described in 25 Indian isolates with high levels of resistance to myclobutanil and cross-resistance to difenoconazole and tetraconazole [145], and also in 39 isolates from Hungary [155]. In addition, other authors described an additional amino acid change (A1119C), which was associated with the increased expression of the *cyp51* gene [21,22].

### 3.8. Resistance to SDHIs in E. necator and P. xanthii

In relation to the new compounds launched on the market, the SDHIs (FRAC code 7) are the class with the fastest growth [156]. In contrast to the limited diseases and application spectrum of the “first-generation” SDHIs (known as carboxamide fungicides), to date, twenty-three SDHI active ingredients belonging to eleven chemical classes with a broader spectrum of fungal activity are available for the control of fungal plant pathogens [78]. Regarding the control of powdery mildews, 11 SDHIs are currently available (Table 1).

The only studies that have shown SDHI resistance problems have been conducted in cucurbit and grape powdery mildews. The first report of SDHI control failure was documented in *P. xanthii* in New York (USA) in 2005 [134] (Table 2). In this study, boscalid only controlled 56% of the *P. xanthii* isolates tested; however, the efficacy of boscalid was almost recovered one year later [134]. Other studies conducted in the same country in the states of Georgia, New York, New Jersey and South Carolina also reported boscalid-resistant *P. xanthii* isolates [50,157,158]. In Japan, resistance to boscalid was also observed, with 34 out of 74 isolates analysed with MIC values of >50 mg/L of the formulated product and 21 of these isolates growing well at 500 mg/L in in vitro bioassays [159]. Another Japanese study also performed showed cross-resistance to boscalid and penthiopyrad but not to fluopyram [160]. Recently, 127 Japanese isolates were tested to check their sensitivity to the commercial fungicides isofematid, isopyrazam, penthiopyrad and pyraziflumid [161]. The results showed that 42.5% of the isolates had moderate levels of resistance to penthiopyrad (EC_50_ = 335.1 – 787.6 mg/L), isopyrazam (EC_50_ = 77.4–266.5 mg/L) and pyraziflumid (EC_50_ = 9.2–31.2 mg/L); 44% of the isolates had high levels of resistance to penthiopyrad (EC_50_ > 1000 mg/L), isopyrazam (EC_50_ > 1070 mg/L) and pyraziflumid (EC_50_ > 1000 mg/L); finally, only 1.5% showed high levels of resistance to isofatamid (EC_50_ > 2400 mg/L) [161]. With respect to the grape powdery mildew, boscalid provided good control of *E. necator* until 2014, when a report described boscalid-resistant isolates collected from French vineyards that were able to grow at 30 and 100 mg/L of the formulated product [162] (Table 2). Nevertheless, these isolates were inhibited with other SDHIs, such as fluxapyroxad and fluopyram [162].

SDHIs have a single-site mode of action. SDHIs inhibit cell energy production by binding to the ubiquinone-binding pocket of succinate dehydrogenase (complex II), which is part of the tricarboxylic cycle and is linked to mitochondrial electron transfer in the fungal respiration pathway [163]. The target enzyme is formed by four subunits: A, which does not interact with the SDHI fungicides, and B, C and D, which form the ubiquinone binding site [164] (Figure 2). Few studies have been performed to clarify the molecular mechanism of resistance to SDHIs in powdery mildews, and only two reports have analysed the different SDH subunits to determine amino acid changes in the grape powdery mildew (Table 3). In *P. xanthii*, mutations in the SdhC and SdhD subunits were correlated with different levels of resistance to SDHIs [161]. Japanese isolates with moderate levels of resistance to penthiopyrad, isopyrazam and pyraziflumid presented the amino acid change S121P in the SdhD subunit; however, the amino acid changes G151R and G172D in the SdhC subunit and H137R in the SdhD subunit were correlated with high levels of resistance to the same fungicides. An additional mutation (A86V) in the subunit C was also correlated with high levels of resistance to isofetamid [161]. In *E. necator*, the amino acid changes H242R and H242Y in the SdhB subunit were correlated with resistance to boscalid [165] and with moderate levels of resistance to fluopyram and fluxapyroxad [162]. The amino acid change G169D in the SdhC subunit was also associated with a decrease in sensitivity to fluxapyroxad and fluopyram in this pathogen [162].

### 3.9. Resistance to Fungicides with an Unknown Mode of Action in P. xanthii

Cyflufenamid (FRAC code U06) and flutianil (FRAC code U13) are active ingredients that belong to a varied group of fungicides with an unknown mode of action (Table 1). Cyflufenamid was registered for powdery mildew control on fruits, vegetables and cereals in Japan, the UK, Europe and the US in 2002, 2005, 2010 and 2012, respectively [166]. Flutianil was registered in 2013 to control cucurbit powdery mildew. The only reports of a reduction in the control efficacy of cyflufemanid and flutianil have been made in the cucurbit powdery mildew.

For cyflufenamid, *P. xanthii* isolates collected from melon and zucchini fields in Italy were uncontrolled with this fungicide [167] (Table 2). It was suspected that, in these fields, the fungicide was applied above the recommended field rate over a few years [167]. In the US, a study performed in the state of Ohio in 2016 showed that cyflufenamid was the less effective fungicide [168]. A year later, 73% and 46% of the *P. xanthii* isolates collected from several cucurbit fields in the same country showed a reduced sensitivity, being able to develop at 10 mg/L and tolerate 50 mg/L of this commercial fungicide, respectively [169]. In addition, the isolates showed resistance to other non-related chemical groups, such as the aryl-phenyl-ketone metrafenone, the aza-naphthalene quinoxyfen, the DMI fungicide myclobutanil, the QoI fungicides and SDHI fungicide boscalid [169].

Regarding flutianil, the first case of resistance was documented in Japan [33] (Table 2). In this study, 89 out of 122 *P. xanthii* isolates were highly resistant to this fungicide, exhibiting EC_50_ values of 100 mg/L of the formulated product and RF of >375.000. Flutianil resistance remained stable after 46 subcultures of the isolates. In addition, cross-resistance to benzoylpyridine pyriofenone was also suggested [33].

## 4. Conclusions and Recommendations

Fungicide resistance is an important agricultural problem that growers face every year. A large amount of money is invested annually in fungicide spray programs, and profits are reduced when resistance arises. A recent publication has described, for example, general costs of 45.14 USD/ha for ground application and 51.80 USD/ha for aerial application in corn in the U.S. [170] or loss of GBP 170 m/year for UK growers to control the cereal eyespot (*Oculimacula yallundae*) during the 1980s [171]. Moreover, the most common powdery mildew fungi are high-risk pathogens, and as documented in this review, important cases of practical resistance have been reported in several parts of the world, such as Africa, Australia, Brazil, Canada, China, Europe (Austria, Belgium, Czech Republic, Denmark, France, Germany, Greece, Hungary, Italy, Portugal, Scandinavia, Spain and the Netherlands), Egypt, India, Iran, Japan, New Zealand, UK, and the US (California, Georgia, Michigan, New Jersey, New York, North Carolina, Ohio, South Carolina and Virginia). In conclusion, the MBC and QoI fungicides have been the chemical classes with more resistance problems in powdery mildew fungi. With respect to QoI fungicides, high EC_50_ values and maintenance of resistance have been observed for several years in different species of powdery mildew fungi. In relation to MBC fungicides, loss of disease control in the field has also been extensively documented. In contrast, chemical classes, such as aza-naphthalenes, are a good option to be included in fungicide spraying programs because they have only had sporadic cases of reduced sensitivity. On the other hand, the molecular mechanisms of resistance have not been elucidated for this chemical group and others, such as aryl-phenyl-ketones hydroxy(-2-amino) pyrimidines and phosphorothiolates, which is in contrast to the body of knowledge regarding resistance to the most popular chemical classes, the MBC, QoI, SBI and SDHI fungicides, being the main mechanism of resistance due to several point mutations in the corresponding target genes.

To manage resistance development, several precautions must be taken with a commercially available fungicide to extend its shelf life as much as possible. The emergence of resistance depends on a number of factors, among others, the structural class of the fungicide and its mode of action play an important role. Those fungicides which have a single mode of action are most likely to develop resistance [19] but, in addition, there is also what is considered a “pathogen risk”, which includes those pathogens with a shorter life cycle (with shorter generation times), abundance of sporulation and the ability of spores to spread, the ability to infect in all the stages and to mutate the fungicide target genes [172]. In addition, insufficient application rate, inherently low efficacy of the fungicide, improper timing or application method and excessive rainfall can also be causes of poor disease control. These factors partly explain why the use of an Integrated Pest Management (IPM) programme is highly recommended. An IPM programme includes the combination of chemical control, the use of resistant crop varieties, biological control agents and appropriate cultural practices, such as the removal of plant debris and crop rotation. Therefore, to reduce the risk of fungicide resistance in powdery mildew fungi, the following recommendations should be considered [17]:

1. Use IPM that includes non-chemical methods.
2. Do not use a single active ingredient; it is highly recommended to use mixtures or more than one fungicide in the fungicide programme.
3. Do not exclusively use a group of fungicides with the same mode of action. It is recommended to use fungicides with at least three different mechanisms of action.
4. In the event that a high-risk fungicide must be used, use it only once per crop, preferably in a mixture with organic multi-site fungicides and other alternatives such as inorganic fungicides (copper and sulphur), host plant defence inductors or biologicals products with multiple modes of actions.
5. Restrict the number of fungicide sprays applied per season and apply them only when strictly necessary, employing different fungicides before and after the season.
6. Use the recommended field doses labelled in the commercial product. Sublethal doses, even in mixtures, favour the appearance of resistance.
7. Achieve good spray coverage (reduces populations exposed to selection).

In addition, as a final recommendation, an important effort should be invested in monitoring fungicide resistance, that is, testing samples of field populations of a target pathogen for its degree of sensitivity to one or more fungicides [173]. Monitoring can be performed to gain early warning of an impending resistance situation and to check whether that management strategy is working [16]. A good guide to perform fungicide monitoring studies with obligate pathogens was reported by Corio-Coset [173]. The cornerstone of monitoring remains some form of bioassay, such that a decrease in sensitivity is identified regardless of the underlying mechanism. However, bioassays for obligate parasites, such as powdery mildews, are very resource-demanding because only leaf disc assays are possible. To avoid these tests, when molecular mechanisms of resistance are known (e.g., a point mutation), various PCR technologies can be applied to detect single nucleotide polymorphisms (SNPs). One of the early examples of these technologies was the large-scale, high-throughput monitoring of QoI resistance in *Blumeria graminis* f. sp. *tritici* using allele-specific real-time PCR [103]. Currently, DNA amplification using isothermal conditions has gained increasing attention because these reactions can be run with less effort and expense compared with PCR and provide quick and reliable results, without the need for sophisticated laboratory equipment [174]. One of the latest examples was the monitoring of MBC resistance in *P. xanthii* using loop-mediated isothermal amplification (LAMP) [74]. Clearly, to manage fungicide resistance in powdery mildews, these techniques must be utilized on a massive scale in crops prone to these diseases.

## Figures and Tables

**Table 1:** Chemical classes of organic fungicides currently registered to control powdery mildew diseases.

Mode of Action	Target	Group Name	Active Ingredient	Authorized Countries ^1^	FRAC Code
**1- MULTI-SITE INHIBITORS**					
Multi-site activity	Multi-site contact activity	Chloronitriles	Chlorothalonil	BG, BR, CA, CL, CO, CR, CZ, EE, FR, IE, IL, JP, LV, LT, MA, MX, NL, PL, RS, RU, SI, SK, TN, TR, UA, UY, VN	M05
		Dithiocarbamates and relatives	Mancozeb	BR, CO, HR, EC, IN, JP, NL, PE, PK, TR, UA, UZ, VN	M03
			Metiram	BG, BR, CZ, FR, HR, IT, MD, PT, RO, SI, SK, TR, UA, ZA	
			Propineb	KZ, VN	
			Zineb	VN	
			Ziram	AR	
		Phthalimides	Captan	MX, UA	M04
			Folpet	AR, BE, BR, BG, CA, CH, CL, DK, EE, ES, FR, GR, HR, IT, LT, MD, MX, NL, PL, PT, RO, RS, SK, TR, UA, UY, VN	
**2- SITE-SPECIFIC FUNGICIDES**					
Amino acids and protein synthesis	Methionine biosynthesis (proposed *cgs* gene)	Anilinopyrimidines	Cyprodinil	AT, BE, BG, CA, CH, CL, CZ, DE, FR, IE, JP, NL, PE, PL, PT, RU, SK, UA, ZA	9
			Mepanipyrim	BE, CL, JP, NL	
			Pyrimethanil	CA, CL, MD, PE, RU, UA	
Cytoskeleton and motor protein	Actin/myosin/fimbrin function	Aryl-phenyl-ketones	Metrafenone	AT, BE, BG, BY, CA, CH, CL, CZ, DE, EC, EE, ES, FR, GR, HR, IN, IE, IL, IT, LT, LV, MD, MA, NL, PE, PL, PT, RO, RU, SK, SI, TN, TR, UA, ZA	50
			Pyriofenone	AT, BE, BG, CA, CL, DE, EE, ES, FI, FR, GR, HR, HU, IE, IL, IT, JP, LV, LT, MD, NL, PE, PL, PT, RS, SI, TR	
	ß-tubulin assembly in mitosis	Methyl-Benzimidazole Carbamates (MBC fungicides)	Benomyl	CL, CR, JP, MX, PE, RU, UA, UZ, ZA	1
			Carbendazim	AR, BR, BY, CL, CR, IN, MX, PE, PK, PY, RU, TN, UA, VN, ZA	
			Fuberidazole	DE	
			Thiabendazole	BY, MD, PE, RU, UA, UY	
			Thiophanate	PK	
			Thiophanate-methyl	AR, BG, BR, BY, CL, CZ, ES, HR, FR, ID, IN, IT, JP, LT, LV, MA, MD, MX, NL, PE, PK, PL, RO, RS, RU, SI, SK, TN, TR, TZ, UA, UY, ZA	
Membrane integrity or function	Phospholipid biosynthesis, methyltransferase	Dithiolanes	Isoprothiolane	PE	6
Nucleic acid metabolism	Adenosin-deaminase	Hydroxy-(2-amino) pyrimidines	Bupirimate	CH, EC, ES, FR, GR, HR, IL, IT, MA, NL, PK, PE, PT, TN, TR, ZA	8
Respiration	Complex II: succinate-dehydrogenase (SdhB, SdhC and SdhD)	Succinate dehydrogenase inhibitors (SDHIs)	Benzovindiflupyr	CA, CH	7
			Bixafen	AT, BE, BR, CH, CL, CZ, DE, EE, ES, FI, FR, IE, IT, LT, LV, MD, NL, PL, PT, SI, SK, TR, UA, ZA	
			Boscalid	AR, AT, BE, BG, CA, CH, CL, CZ, DE, DK, EC, EE, ES, FR, HR, IE, IL, IN, IT, LT, LV, MA, MD, NL, NZ, PE, PL, PT, SI, SK, TN, TR, UA, UY, ZA	
			Carboxin	BY, UA	
			Fluopyram	AT, BE, BG, BY, CA, CH, CL, CR, CZ, DE, EE, ES, FI, FR, GR, HR, HU, IE, IL, IN, IT, JP, LT, LV, MA, MD, NL, PE, PL, PT, RS, RU, SI, SK, TN, TR, UA, ZA	
			Fluxapyroxad	AT, BE, BY, BG, CA, CH, CL, CZ, DE, EE, ES, FR, GR, IE, IT, LT, LV, MD, NL, NZ, PL, PT, RU, RS, SI, SK, TN, TR, UA, ZA	
			Isofetamid	JP	
			Isopyrazam	AT, BE, BG, CL, CZ, DE, ES, GR, HR, IE, IT, JP, NL, PL, PT, RS, RU, SI, SK, TR, UA	
			Penthiopyrad	BG, BY, CH, CZ, GR, JP, NL, PL, RU, UA	
			Pydiflumetofen	NZ	
			Pyraziflumid	JP	
			Sedaxane	UK	
	Complex III: cytochrome bc1 at Qo site (Cytb)	Quinone outside inhibitors (QoIs)	Azoxystrobin	AR, AT, BE, BR, BG, BY, CA, CH, CL, CZ, DE, EC, EE, ES, FI, FR, DE, GR, HR, HU, IN, IE, IL, IN, IT, JP, LT, LV, MA, MD, MY, MX, NL, PE, PL, PT, RU, RS, SK, SI, TN, TR, UA, UY, ZA	11
			Dimoxystrobin	PL, SK	
			Famoxadone	BG	
			Fluoxastrobin	AT, BE, BY, CA, CH, CL, CZ, DE, EC, EE, FR, IE, LT, LV, NL, PL, RU, SK, TR, UA	
			Kresoxim-methyl	AR, AT, BE, BG, BR, BY, CH, CL, CZ, DE, EC, EE, ES, FR, GR, HR, IE, IL, IN, IT, JP, LT, LV, MA, MD, MY, NL, PE, PL, PT, PY, RU, RS, SI, SK, TN, TR, UA, UY, VN, ZA	
			Mandestrobin	CA, JP	
			Metominostrobin	IL, PE	
			Picoxystrobin	BR, BY, CA, CH, IL, IN, RU, TR, UA, ZA	
			Pyraclostrobin	AR, AT, BE, BG, BO, BR, BY, CA, CH, CL, CZ, DE, EE, ES, FI, FR, GR, HR, IE, IL, IN, IT, LT, LV, MD, NL, NZ, PE, PL, PT, RO, RS, RU, SI, SK, TN, TR, UA, UY, ZA	
			Trifloxystrobin	AR, AT, BE, BG, BR, CA, CH, CL, CR, CZ, DE, EC, ES, FR, GR, HR, HU, IE, IL, IN, IT, MA, MD, MX, NL, PE, PK, PL, PT, PY, RS, RU, SI, SK, TN, TR, UA, UY, VN, ZA	
	Uncouplers of oxidative phosphorylation	2,6-Dinitro-anilines	Fluazinam	BR, CH	29
		Dinitrophenyl-crotonates	Dinocap	IN, MX	
			Meptyldinocap	AT, CL, CZ, ES, FR, GR, HR, IL, IT, MA, PE, PT, RS, SK, SI, TN, TR	
Signal transduction	Signal transduction (mechanism unknown)	Aza-naphthalenes	Quinoxyfen	AR, CA, CH, CL, ES, IL, MA, NZ, PE, PT, RS, TR, UA, ZA	13
			Proquinazid	AT, BG, BY, CL, CH, CZ, DE, EE, ES, FI, FR, GR, HR, IE, IL, IT, LT, LV, MA, MD, PE, PL, PT, RO, RU, SE, SI, SK, TN, TR, UA, ZA	
	MAP/Histidine-Kinase in osmotic signal transduction (Os-2, HOG1)	Phenylpyrroles (PP-fungicides)	Fludioxonil	BG, CL, FR, LT, PT, RU, UA, ZA	12
Sterol biosynthesis in membranes	Δ^14^-reductase and Δ^8^→Δ^7^-isomerase (Erg24, Erg2)	Amines (“morpholines”)	Fenpropidin	AT, BE, BG, BY, CH, CL, CZ, DE, EE, FR, IE, IT, LT, LV, MD, NL, PL, SK, UA	5
			Fenpropimorph	AT, BE, BG, BY, CH, CL, CZ, DE, EE, FR, IE, LT, LV, MD, NL, PL, RO, RU, SI, SK, TR, UA	
			Spiroxamine	AT, BE, BG, BY, CA, CH, CL, CZ, DE, EC, EE, ES, FR, HR, HU, IE, IL, IT, LT, LV, MD, NL, PE, PL, PT, RO, RS, RU, SI, SK, TN, TR, UA, ZA	
			Tridemorph	PK	
	C14- demethylase (Erg11/Cyp51)	Demethylation inhibitors (DMI-fungicides)	Bromuconazole	AT, BE, CZ, DE, NL, PE, PL, TN, TR, UA	3
			Cyproconazole	AR, AT, BE, BG, BO, BR, BY, CH, CL, CZ, DE, EE, ES, FI, FR, GR, HR, IL, IT, LT, LV, MA, MD, NL, NZ, PE, PL, PY, RS, RU, SI, SK, TN, TR, UA, UY, UZ, ZA	
			Difenoconazole	AR, AT, BE, BR, BG, BY, CA, CH, CL, CZ, DE, EC, EE, ES, FR, GR, HR, IN, IL, IT, JP, LT, LV, MA, MD, NL, NZ, PK, PE, PL, PT, RU, RS, SI, SK, TN, TR, UA, UY, UZ, ZA	
			Diniconazole	PE	
			Epoxiconazole	AR, AT, BE, BG, BO, BR, BY, CH, CL, CZ, DE, DK, EE, ES, FR, GR, HR, IE, IT, LT, LV, MD, NL, PL, PY, RO, RS, RU, SI, SK, TN, TR, UA, UY, ZA	
			Fenarimol	CL, JP, PK, PE	
			Fenbuconazole	BG, CL, HR, FR, GR, IL, IT, JP, PT, ES, TR	
			Fluquinconazole	CL	
			Flusilazole	BG, TR, ZA	
			Flutriafol	BR, BG, BY, CA, CL, CZ, ES, FR, GR, HR, IE, IT, MD, MX, PY, PE, PL, PT, RU, RS, TN, UA, UY, UZ, ZA	
			Hexaconazole	CO, IN, MA, MX, PK, PE, VN, ZA	
			Imazalil	BE, BY, DE, MD, NL, RU, UA	
			Imibenconazole	BR, JP, VN	
			Ipconazole	UA	
			Mefentrifluconazole	AT, CA, CZ, DE, EE, FR, LT, LV, PE, PL, TR	
			Metconazole	AR, AT, BE, BG, BR, BY, CA, CH, CL, CZ, DE, EE, FI, FR, HR, IE, IT, LT, MD, PL, PT, PY, RS, RU, SI, SK, UA, UY	
			Myclobutanil	AR, AT, BE, BR, BG, CA, CH, CL, CR, CZ, DE, EC, ES, FR, GR, HR, IL, IN, IT, JP, MA, MD, MX, PE, PT, RO, RS, SI, SK, TN, TR, UA, ZA	
			Penconazole	AR, AT, BE, BG, BY, CH, CL, CZ, DE, EE, ES, FR, GR, HR, IL, IN, IT, LT, LV, MA, MD, NL, PE, PK, PL, PT, RS, RU, SI, SK, TN, TR, UA, UZ, ZA	
			Prochloraz	AT, BE, BG, BY, CH, CL, CZ, DE, EE, ES, FR, GR, HR, IE, IT, LT, LV, MD, MX, NL, PE, PL, TR, RU, SK, TR, UA, ZA	
			Propiconazole	BG, BO, BR, BY, CA, CH, CL, CO, IN, JP, MX, PE, PK, RS, RU, TN, TR, UA, UY, UZ, ZA	
			Prothioconazole	AT, BE, BG, BR, BY, CA, CH, CL, CZ, DE, DK, EE, ES, FI, FR, GR, HR, HU, IE, IT, LT, LV, MD, NL, PL, PT, RS, RU, SI, SK, TN, TR, UA, UY, ZA	
			Simeconazole	JP	
			Tebuconazole	AR, AT, BE, BG, BR, BY, CA, CH, CL, CZ, DE, DK, EC, EE, ES, FI, FR, GR, HR, HU, IE, IL, IN, IT, JP, LT, LV, MA, MD, MX, NL, PE, PL, PT, PY, RO, RU, RS, SI, SK, TN, TR, UA, UY, UZ, ZA	
			Tetraconazole	AT, BE, BG, BR, CA, CL, CZ, DE, ES, FR, GR, HR, IL, IT, MA, MD, PE, PL, PT, RU, SI, SK, TR, UA, ZA	
			Triadimefon	AR, BY, CL, CR, IN, KE, MX, RU, TZ, UA, UZ, ZA	
			Triadimenol	AR, BR, BG, CH, CL, CZ, DE, EC, EE, FR, GR, HR, IL, IT, LT, LV, MA, MD, NZ, PE, PL, RO, RS, RU, SI, SK, TN, TR, UA, ZA	
			Triflumizole	BE, BR, CL, JP, MA, MD, MX, NL, PE	
			Triforine	CL, JP, MX, PK, UZ	
			Triticonazole	BR, BY, CL, MD, RU, UA, ZA	
**3- UNKNOWN MODE OF ACTION**					
Unknown	Unknown	Phenyl-acetamide	Cyflufenamid	AT, BR, BG, CH, CL, CZ, DE, EE, ES, FR, GR, HR, IN, IE, IL, IT, JP, LT, LV, MA, NL, PE, PL, PT, RS, RU, SI, SK, TN, TR, UA, ZA	U06
		Thiazolidine	Flutianil	JP	U13

^1^ Active ingredients registered in several countries such as Argentina (AR), Austria (AT), Belarus (BY), Belgium (BE), Bolivia (BO), Brazil (BR), Bulgaria (BG), Canada (CA), Chile (CL), China (CN), Colombia (CO), Costa-Rica (CR), Croatia (HR), Czech Rep. (CZ), Denmark (DK), Ecuador (EC), Estonia (EE), Finland (FI), France (FR), Germany (DE), Greece (GR), Hungary (HU), India (IN), Indonesia (ID), Ireland (IE), Israel (IL), Italy (IT), Japan (JP), Kazakhstan (KZ), Kenya (KE), Latvia (LV), Lithuania (LT), Malaysia (MY), Mexico (MX), Moldova (MD), Morocco (MA), Netherlands (NL), New Zealand (NZ), Pakistan (PK), Paraguay (PY), Peru (PE), Poland (PL), Portugal (PT), Romania (RO), Russian Fed. (RU), Serbia Rep. (RS), Slovakia (SK), Slovenia (SI), South-Africa (ZA), Spain (ES), Sweden (SE), Switzerland (CH), Tanzania (TZ), Tunisia (TN), Turkey (TR), Ukraine (UA), Uruguay (UY), Uzbekistan (UZ) and Vietnam (VN). The data were kindly provided by Lexagri SAS (ex-Agrobase-logigram) database.

**Table 2:** Occurrence of field fungicide resistance in powdery mildews.

Group Name	Active Ingredient	Year of Registration ^1^	Resistant Pathogen	Date First Observed	Reference
Aryl-phenyl ketones	Metrafenone	2006	*B. graminis* f. sp. *tritici*	2009	[27]
			*E. necator*	2010	[32]
	Pyriofenone	2014	*P. xanthii*	2017	[33]
Aza-naphthalenes	Quinoxyfen	1996	*E. necator*	2002	[42]
			*B. graminis* f. sp. *tritici*	2003	[38]
			*P. xanthii*	2004	[49]
Hydroxy-(2-amino) pyrimidines	Dimethirimol	1968	*P. xanthii*	1970	[55]
	Ethirimol	1968	*B. graminis* f. sp. *hordei*	1971	[57]
	Bupirimate	1975	*P. xanthii*	1986	[60]
			*G. cichoracearum*	1990	[62]
Methyl-Benzimidazole Carbamates (MBC fungicides)	Benomyl	1968	*P. xanthii*	1967	[66]
			*S. pannosa*	1974	[75]
			*E. necator*	1977	[76]
			*G. cichoracearum*	2001	[70]
	Thiophanate-methyl	1971	*P. xanthii*	2002	[64]
			*E. necator*	2005	[46]
			*G. cichoracearum*	2005	[73]
Phosphorothiolates	Pyrazophos	1970	*P. xanthii*	1975	[81]
Quinone outside Inhibitors (QoI-fungicides)	Azoxystrobin	1992	*P. xanthii*	1998	[86]
			*E. necator*	1999	[89]
			*G. cichoracearum*	2010	[73]
			*L. taurica*	2015	[99]
	Kresoxim-methyl	1996	*P. xanthii*	1998	[86]
			*B. graminis* f. sp. *tritici*	1998	[87]
			*B. graminis* f. sp. *hordei*	2003	[88]
	Famoxadone	1998	*B. graminis* f. sp. *tritici*	2000	[83]
			*P. xanthii*		
	Trifloxystrobin	1999	*B. graminis* f. sp. *tritici*	1998	[87]
			*E. necator*	2002	[90]
			*P. leuchotricha*	2002	[98]
			*P. xanthii*	2002	[84]
			*B. graminis* f. sp. *hordei*	2003	[88]
			*E. betae*	2011	[96]
	Pyraclostrobin	2000	*E. betae*	2011	[96]
	Fenamidone	2001	*B. graminis* f. sp. *tritici*	2000	[83]
			*P. xanthii*		
Amines (“morpholines”)	Tridemorph	1969	*B. graminis* f. sp. *hordei*	1976	[58]
			*P. xanthii*	1988	[61]
	Fenpropimorph	1983	*B. graminis* f. sp. *hordei*	1979	[109]
	Fenpropidin	1986	*B. graminis* f. sp. *hordei*	1979	[109]
	Spiroxamine	1997	*E. necator*	2002	[77]
DeMethylation Inhibitors (DMI-fungicides)	Triforine	1970	*P. xanthii*	1982	[128]
	Fenarimol	1975	*P. xanthii*	1984	[129]
			*E. necator*	1985	[138]
			*G. cichoracearum*	2005	[73]
	Triadimefon	1976	*B. graminis* f. sp. *hordei*	1979	[110]
			*B.graminis* f. sp. *tritici*	1981	[112]
			*E. necator*	1985	[138]
			*P. xanthii*	1988	[61]
	Imazalil	1977	*P. xanthii*	1982	[128]
	Prochloraz	1977	*B.graminis* f. sp. *tritici*	1981	[112]
			*B. graminis* f. sp. *hordei*		
	Triadimenol	1978	*B. graminis* f. sp. *hordei*	1979	[110]
			*B.graminis* f. sp. *tritici*	1981	[112]
			*E. necator*	1990	[139]
			*P. xanthii*	2002	[133]
	Diclobutrazol	1979	*B.graminis* f. sp. *tritici*	1981	[112]
			*B. graminis* f. sp. *hordei*		
	Propioconazole	1980	*B.graminis* f. sp. *tritici*	1981	[112]
			*B. graminis* f. sp. *hordei*		
			*P. xanthii*	1991	[67]
	Nuarimol	1980	*B.graminis* f. sp. *tritici*	1981	[112]
			*B. graminis* f. sp. *hordei*		
	Flutriafol	1981	*B. graminis* f. sp. *hordei*	1992	[116]
	Penconazole	1983	*P. xanthii*	1988	[61]
			*E. necator*	1996	[141]
			*P. aphanis*	2009	[148]
	Flusiazole	1984	*E. necator*	1996	[141]
	Cyproconazole	1986	*B. graminis* f. sp. *hordei*	1991	[119]
	Hexaconazole	1986	*E. necator*	2009	[146]
	Myclobutanil	1986	*E. necator*	1985	[138]
			*P. xanthii*	1991	[67]
			*P. aphanis*	2009	[148]
	Tebuconazole	1986	*B. graminis* f. sp. *hordei*	1992	[116]
			*E. necator*	2004	[90]
	Triflumizole	1986	*E. necator*	2004	[90]
	Difenoconazole	1988	*P. xanthii*	2008	[132]
	Epoxiconazole	1993	*B. graminis* f. sp. *hordei*	1991	[119]
Succinate DeHydrogenase Inhibitors (SDHI-fungicides)	Boscalid	2002	*P. xanthii*	2005	[134]
			*E. necator*	2014	[164]
	Penthiopyrad	2010	*P. xanthii*	2017	[161]
	Isopyrazam	20172017			
	Pyraziflumid				
	Isofetamid	2018		2018	
Phenyl-acetamide	Cyflufenamid	2002	*P. xanthii*	2012	[167]
Cyano-methylenethiazolidines	Flutianil	2008	*P. xanthii*	2017	[33]

^1^ Acording to the Pesticide Propierties DataBase of the University of Hertfordshire (https://sitem.herts.ac.uk/aeru/footprint/es/atoz.htm).

**Table 3:** Amino acid changes in target proteins and other molecular mechanisms related with fungicide resistance to MBC fungicides, QoIs, DMIs and SDHIs in powdery mildews.

Group Name	Active Ingredient	Resistant Pathogen	Target Protein	Amino Acid Change	Phenotype	References	
Methyl-Benzimidazole Carbamates (MBC fungicides)	Thiophanate-methyl	*P. xanthii*	β-tubulin	E198A	Highly resistant	[74,80]	
Quinone outside Inhibitors (QoI-fungicides)	Azoxystrobin Kresoxim-methyl Pyraclostrobin Tryfloxystrobin	*B. graminis* f. sp. *hordei*	Cytb	G143A	Resistant	[88]	
		*B. graminis* f. sp. *tritici*				[83,101,102,103,104,105]	
		*E. betae*				[96,97]	
		*E. necator*				[46,91,92,93,94,95]	
		*L. taurica* ^1^				[99]	
		*P. leucotricha* ^1^				[98]	
		*P. xanthii* ^1^				[82,83,106]	
DeMethylation Inhibitors (DMI-fungicides)	Propiconazole	*B. graminis* f. sp. *tritici*	Cyp51	Y136F, K147Q	Resistant	[150]	
	Triadimenol						
	Epoxiconazole	*B. graminis* f. sp. *hordei*	Cyp51	Y136F, S509T	Resistant	[153]	
	Tebuconazole			Y136F, S509T	Resistant	[151,152,153]	
	Triadimenol			Y136F, K147Q	Highly-low resistant	[149,150]	
	Fenarimol	*E. necator*	Cyp51	Y136F, A1119C ^2^	Resistant, highly resistant	[22]	
	Myclobutanil			Y136F, A1119C ^2^	Highly resistant	[21,22,145]	
	Tebuconazole			Y136F, A1119C ^2^	Resistant, highly resistant	[22]	
	Triadimenol			Y136F	Highly resistant	[154]	
Succinate DeHydrogenase Inhibitors (SDHI-fungicides)	Boscalid	*E. necator*	SdhB	H242R/Y	Resistant	[165]
	Fluopyram Fluxapyroxad	*E. necator*	SdhB	H242R/Y	Moderately resistant	[31]
			SdhC	G169D	Highly resistant	
	Penthiopyrad Isopyrazam Pyraziflumid	*P. xanthii*	SdhC	G151R	Highly resistant	[161]
				G172D		
			SdhD	H137R		
				S121P	Moderately resistant	
	Isofetamid		SdhC	A86V	Highly resistant	

^1^ Heteroplasmy described. ^2^ The silent mutation A1119C increases the expression of *cyp51* gene.
